# Hypoxia enhances the expression of autocrine motility factor and the motility of human pancreatic cancer cells

**DOI:** 10.1038/sj.bjc.6600331

**Published:** 2002-06-17

**Authors:** H Niizeki, M Kobayashi, I Horiuchi, N Akakura, J Chen, J Wang, J-i Hamada, P Seth, H Katoh, H Watanabe, A Raz, M Hosokawa

**Affiliations:** Division of Cancer Pathobiology, Institute for Genetic Medicine, Hokkaido University, Kita-15, Nishi-7, Kita-ku, Sapporo 060-0815, Japan; Division of Cancer-Related Genes, Institute for Genetic Medicine, Hokkaido University, Sapporo 060-0815, Japan; Division of Gene Therapy Development, Institute for Genetic Medicine, Hokkaido University, Sapporo 060-0815, Japan; Department of Surgical Oncology, Hokkaido University Graduate School of Medicine, Hokkaido Japan; Department of Gastroenterology and Hematology, Hokkaido University Graduate School of Medicine, Sapporo, Hokkaido 060-8638, Japan; Department of Orthopedic Surgery, Gunma University, Showa 3-39-29, Maebashi, 371-8511, Japan; Division of Basic Research, Karmanos Cancer Institute, Wayne State University School of Medicine, Detroit, Michigan, MI 4820, USA

**Keywords:** hypoxia, AMF, motility, HIF-1, metastasis

## Abstract

The incidence of distant metastases is higher in the tumours with low oxygen pressure than in those with high oxygen pressure. It is well known that hypoxia induces the transcription of various genes involved in angiogenesis and anaerobic metabolism necessary for the growth of tumour cells *in vivo*, suggesting that hypoxia may also induce the transcription of metastasis-associated genes. We sought to identify the metastasis-associated genes differentially expressed in tumour cells under hypoxic conditions with the use of a DNA microarray system. We found that hypoxia enhanced the expression of autocrine motility factor mRNA in various cancer cells and also enhanced the random motility of pancreatic cancer cells. Autocrine motility factor inhibitors abrogated the increase of motility under hypoxic conditions. In order to explore the roles of hypoxia-inducible factor-1α, we established hypoxia-inducible factor-1α-transfectants and dominant negative hypoxia-inducible factor-1α-transfectants. Transfection with hypoxia-inducible factor-1α and dominant-negative hypoxia-inducible factor-1α enhanced and suppressed the expression of autocrine motility factor/phosphohexase isomerase/neuroleukin mRNA and the random motility, respectively. These results suggest that hypoxia may promote the metastatic potential of cancer cells through the enhanced autocrine motility factor/phosphohexase isomerase/neuroleukin mRNA expression and that the disruption of the hypoxia-inducible factor-1 pathway may be an effective treatment for metastasis.

*British Journal of Cancer* (2002) **86**, 1914–1919. doi:10.1038/sj.bjc.6600331 www.bjcancer.com

© 2002 Cancer Research UK

## 

As metastasis is the major cause of death in cancer patients, control of metastasis is most important in the therapies for cancer patients. In order to control metastasis, we need to understand the details of metastatic process that is now thought to take multiple steps. Although a variety of factors have been documented to play important roles in the metastatic steps (Poste and Fidler, 1980; Liotta, 1986, 1988; Nicolson, 1988), many factors are yet to be elucidated. Several clinical investigations demonstrated that patients with hypoxic tumours had poor prognoses and that the incidence of distant metastases was higher in the tumours with low oxygen pressure than in those with high oxygen pressure (Gatenby *et al*, 1988; Brizel *et al*, 1996; Hockel *et al*, 1998; Rofstad, 2000). These studies suggest that the tumour cells exposed to hypoxia at the primary tumour sites acquire aggressive properties including metastatic potential more than the well-oxygenated tumour cells do. In fact, recent reports have demonstrated that hypoxia enhances the expression of vascular endothelial growth factor (VEGF) and interleukin-8 (IL-8) in tumour cells, resulting in an increase of metastatic potential (Claffey and Robinson, 1996; Shi *et al*, 1999; Biroccio *et al*, 2000). However, it remains poorly understood how hypoxia promotes tumour cells' metastatic potential.

When tumour cells are exposed to hypoxia, hypoxia-inducible factor-1 (HIF-1), which is a transcription factor composed of HIF-1α and HIF-1β subunits (Wang *et al*, 1995; Wang and Semenza, 1996), is activated and then it promotes the transcription of several genes such as glucose transporters, glycolytic enzymes, and angiogenic factors (Dang and Semenza, 1999). A recent report demonstrates that human common cancer cells over-express hypoxia-inducible factor-1α (HIF-1α) *in vivo* and that the expression of HIF-1α is associated with metastasis (Zhong *et al*, 1999). Furthermore, infiltration of endothelial cells, which involves various factors, is enhanced when tumour tissues are in hypoxia-induced angiogenesis. These reports have prompted us to hypothesise that hypoxia may also promote the invasion and metastasis of tumour cells by promoting the expression of metastasis-associated genes in addition to angiogenic factors through the activation of HIF-1α. As hypovasculature is an outstanding characteristic of pancreatic cancers with high invasiveness and metastatic potential (Raijiman and Levin, 1995) and most pancreatic cancer cells over-express HIF-1α protein (Akakura *et al*, 2001), we speculate that pancreatic cancer cells may be exposed to severe hypoxia *in vivo* and that pancreatic cancers express higher levels of metastasis-associated genes in addition to angiogenic factors than well-oxygenated tumour cells do. Therefore we examined the expression of more than 9000 mRNAs in a pancreatic cancer cell line under hypoxic and non-hypoxic conditions with the use of a DNA microarray system and compared them to identify the metastasis-associated genes induced by hypoxia.

We found that autocrine motility factor (AMF)/phosphohexose isomerase (PHI)/neuroleukin (NL) mRNA was expressed more highly in the cells under hypoxic conditions than under non-hypoxic conditions. In this study, we examined the expression of AMF/PHI/NL mRNA in a variety of cancer cell lines and the random motility of a pancreatic cancer cell line under hypoxic and non-hypoxic conditions, because AMF/PHI/NL was reported to stimulate random motility (Liotta *et al*, 1986). Furthermore, we established HIF-1α-transfectants and dominant-negative HIF-1α-transfectants and examined their expression of AMF/PHI/NL mRNA and random motility in order to determine the possible role of HIF-1α in the AMF/PHI/NL mRNA expression and random motility promoted by hypoxia.

## MATERIALS AND METHODS

### Cell lines

Pancreatic ductal adenocarcinoma cell lines (PCI-6, PCI-10, PCI-19, PCI-43 and PCI-66 cells) were established from surgically resected pancreatic cancer tissues. These cell lines were kindly supplied from Dr Hiroshi Ishikura (The First Department of Pathology, Hokkaido University School of Medicine, Japan) and maintained in DMEM/F12 medium supplemented with 10% foetal calf serum (FCS). TAOV (ovarian cancer), TTOV (ovarian cancer), HepG2 (hepatoma), PC-6 (lung cancer), MiaPaca-2 (pancreatic cancer), BxPC-3 (pancreatic cancer) and KM-12 (colon cancer) cells were maintained in DMEM medium supplemented with 10% FCS.

### DNA microarray analysis

Total RNA was extracted with the use of TRIZOL Reagent (Life Technologies, Tokyo, Japan) from the PCI-10 cells that had been incubated for 24 h under non-hypoxic and hypoxic conditions. The incubation under hypoxic condition (1% O_2_) was done in a hypoxic chamber gassed with 95% N_2_ and 5% CO_2_ (Wakenyaku Co. Ltd., Tokyo, Japan). mRNA was purified from the total RNA with the use of a Quickprep mRNA purification kit (Amersham Pharmacia Biotech, Tokyo, Japan). Differentially expressed genes were screened with the use of a DNA microarray system, UniGEM human (Kurabo Industries Ltd, Osaka, Japan). We defined the genes expressed at more than two-fold higher levels under hypoxic conditions than under non-hypoxic conditions as possible hypoxia-inducible genes in this study.

### Establishment of HIF-1α-transfectants and dominant negative HIF-1α-transfectants

Establishment of HIF-1α-transfectants was previously reported (Akakura *et al*, 2001). A cDNA for dominant negative HIF-1α (dnHIF-1α) lacking both the DNA binding and transactivation domains of HIF-1α was amplified from reverse transcription (RT) products of mRNAs purified from the PCI-10 cells and cloned into PCR4-TOPO (Invitrogen, Carlsbad, CA, USA). PCR primers for dominant negative HIF-1α were selected as previously described (Halterman *et al*, 1999) (forward, ccgctcgagaccatgcgaaggaaagaatctg; reverse, ggggtacctcatttgtcaaagaggctact). Plasmids were recovered, purified and sequenced with a DyeDeoxy Terminator kit (Perkin-Elmer, Urayasu, Japan) on an ABI 377 automated sequencer (Applied Biosystems, Urayasu, Japan) in the condition described in the manufacturer's protocol. Cloned fragments were recovered from the vectors and ligated into PcDNA3.1+ (Invitrogen, Carlsbad, CA, USA). PCI-43 cells were transfected with the expression vector for dnHIF-1α with the use of Lipofectamine (Life Technologies, Tokyo, Japan). Transfectants were cloned by a limiting dilution method after the selection with G-418 at 800 μg ml^−1^. The transfectants were maintained in the presence of 400 μg ml^−1^ of G-418.

### FACS analysis

Cells were stained with anti-AMFR antibody (rat monoclonal antibody, IgM) (Niinaka *et al*, 1998) for 1 h and then stained with a second anti-rat Ig antibody for 1 h. Expression of AMFR proteins was quantified with the use of FACScaliber (Becton Dickinson, Mountain View, CA, USA).

### Northern blot analysis

Northern blot analysis was performed by the method described previously (Choi *et al*, 2000). Total RNA (20 μg) was separated by electrophoresis in 1.2% denaturing formaldehyde-agarose gels. The RNA was transferred to nylon membrane (Hybond N^+^, Amersham) overnight by capillary elution and UV cross-linked. After prehybridisation of the blots for 1–2 h at 42°C in hybridisation buffer (5×SSPE, 5×Denhardt's solution, 1% SDS, 50% Formamide and 0.1 mg ml^−1^ of denatured salmon sperm DNA), the membrane was hybridised overnight at 42°C with the cDNA probes for AMF and AMF receptor (AMFR). The probed membrane was then washed and exposed to radiographic film. cDNA fragments for AMF and AMF receptor (AMFR) were amplified by RT–PCR, cloned into a TA cloning vector, purified from the vector and then used as probes for Northern blot analysis. PCR primers were as follows: AMF forward, gcttgaccctcaacaccaac; reverse, gaagtgctggtccatccagt; AMFR forward, caggaggaagtgagccagtc; reverse, agcttgctgcctaaccactc.

### Phagokinetic track assay

Phagokinetic track assay was performed according to the method reported by Albrecht-Buehler (1977). Briefly, gold particle suspension was prepared by mixing 18 μl of 14.5 mM AuCl_4_ solution and 5 ml of 36.5 mM Na_2_CO_3_ solution with 11 ml double-distilled water followed by heating to boiling point and adding 1.8 ml of 0.1% formaldehyde. Two ml of gold particle suspension was laid on the 18×18 mm-square glass coverslips coated with 0.1% bovine serum albumin. The particle-coated coverslips were placed in 35 mm plastic dishes containing DMEM/F-12 medium supplemented with 5% FCS. Cells were seeded in the dishes and incubated for 24 h. After incubation, the coverslips were removed and fixed in 10% formaldehyde solution for 30 min and mounted on microscope slides. Phagokinetic track areas of more than 20 cells that were randomly selected were observed with the use of a microscope analyser (Cosmo zone R500, Nikon, Japan). The experiments were done under hypoxic (1% O_2_ per 5% CO_2_) and non-hypoxic conditions (20% O_2_ per 5% CO_2_). In some experiments, supernatant of the pancreatic cancer cells PCI-10 cultured in hypoxia was added in the dishes at 0, 12.5, 25, 50 and 100%. PHI inhibitors described in the previous report (Watanabe *et al*, 1996), erythrose 4-phosphate (E4P) and 6-phosphogluconic acid (6PGA) (Sigma) were added into the dishes at 10, 100 and 1000 μM.

### Statistical analysis

Statistical analysis was done by the Mann–Whitney test.

## RESULTS

### Differentially expressed genes under hypoxic conditions

We defined the genes expressed at more than two-fold higher levels under hypoxic conditions than under non-hypoxic conditions as possible hypoxia-induced genes, which numbered 38. As expected, mRNAs for glycolytic enzymes and angiogenic factors were expressed at higher levels under hypoxic conditions than under non-hypoxic conditions. In the glycolytic enzymes, we found that phosphohexose isomerase (PHI) mRNA was expressed at a two-fold higher level under hypoxic conditions than under non-hypoxic conditions. A recent report has demonstrated that AMF, which was originally identified as a molecule stimulating directional motility (chemotaxis) and random motility (chemokinesis) of melanoma cells (Liotta *et al*, 1986), was identical to PHI/NL (Watanabe *et al*, 1996). As AMF/PHI/NL secreted by tumour cells acts as a chemokinetic factor (random motility-stimulating factor) in an autocrine fashion, we focused on this molecule that could promote the metastatic potential under hypoxic conditions.

### Expressions of AMF/PHI/NL and AMFR in a variety of cancer cells

**Figure 1:**
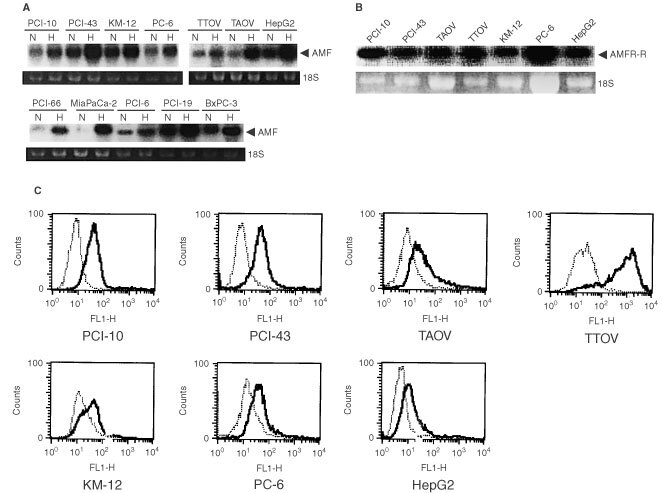
Expression of AMF/PHI/NL and AMFR mRNAs. (**A**) AMF/PHI/NL mRNA expression. RNAs were extracted from the cells incubated under non-hypoxic (N) and hypoxic (H) conditions (20% and 1%, respectively) for 24 h and then Northern blot analysis was performed. (**B**) AMFR mRNA expression. RNAs were extracted from the cells incubated under non-hypoxic (N) for 24 h and then Northern blot analysis was performed. A representative result of two different experiments is shown. (**C**) FACS analysis of AMFR expression. Cells were stained with anti-AMFR antibody (rat monoclonal antibody, IgM) for 1 h and then stained with second anti-rat Ig antibody for 1 h. A representative result of two different experiments is shown.

We then examined the expression of AMF/PHI/NL mRNA in several cancer cell lines, including PCI-10 cells used in the DNA microarray analysis, before and after exposure to hypoxia in order to define whether the enhanced expression of AMF/PHI/NL mRNA in hypoxia was common in a variety of cancer cells. Figure 1A

shows the expression of AMF/PHI/NL mRNA in the pancreatic cancer cell lines (PCI-6, PCI-10, PCI-19, PCI-43, PCI-66, MiaPaca-2 and BxPC-3), colon cancer cell line (KM-12), lung cancer cell line (PC-6), hepatoma cell line (HepG2) and ovarian cancer cell lines (TAOV and TTOV). All the cell lines expressed AMF/PHI/NL mRNA at higher levels under hypoxic conditions than under non-hypoxic conditions, indicating that the increased expression of AMF/PHI/NL mRNA under hypoxic conditions was common in a variety of cancer cells. Next we examined the expression of AMFR. All the cell lines examined, especially PCI-10 cells used for the examination of motility, expressed AMFR mRNA and protein (Figure 1B,C), suggesting that the AMF/PHI/NL could stimulate the random motility of the cells in an autocrine fashion.

### Expression of AMF/PHI/NL mRNA in HIF-1α-transfectants and dominant-negative HIF-1α-transfectants

Figure 2A

**Figure 2:**
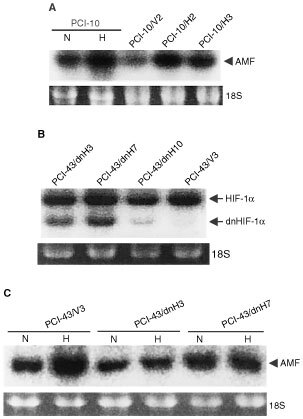
Expression of AMF/PHI/NL mRNA in the transfectants: (**A**) Northern blot analysis of AMF/PHI/NL mRNA in the HIF-1α-transfectants (PCI-10/H2 and PCI-10/H3) and vector-transfectant (PCI-10/V2) under non-hypoxic conditions. AMF/PHI/NL mRNA expression in PCI-10 cells under non-hypoxic (N) and hypoxic (H) conditions were shown as controls. (**B**) Northern blot analysis of dominant negative HIF-1α (dnHIF-1α) mRNA in the transfectants. (**C**) Northern blot analysis of AMF/PHI/NL mRNA expression in the dnHIF-1α -transfectants (PCI-43/dnH3 and PCI-43/dnH7) and the vector-transfectant (PCI-43/vV3) under non-hypoxic (N) and hypoxic (H) conditions. RNAs were extracted from the cells incubated under non-hypoxic (N) and hypoxic (H) conditions for 24 h and then Northern blot analysis was performed.

shows the expression of AMF/PHI/NL mRNA in the HIF-1α-transfectants and the vector-transfectant under non-hypoxic conditions. The expression of AMF/PHI/NL mRNA was higher in the HIF-1α-transfectants than in the vector-transfectant. Figure 2B shows the expression of dominant-negative HIF-1α (dnHIF-1α) mRNA. The dnHIF-1α-transfectants but not the vector-transfectant expressed the truncated dnHIF-1α mRNA in addition to the endogenous HIF-1α mRNA. Expression of AMF/PHI/NL mRNA was enhanced after exposure to hypoxia in the vector-transfectant and the parent cells but not in the dnHIF-1α-transfectants (Figure 2C). These results suggested that hypoxia might enhance the expression of AMF/PHI/NL mRNA through the activation of HIF-1.

### Random motility

**Figure 3:**
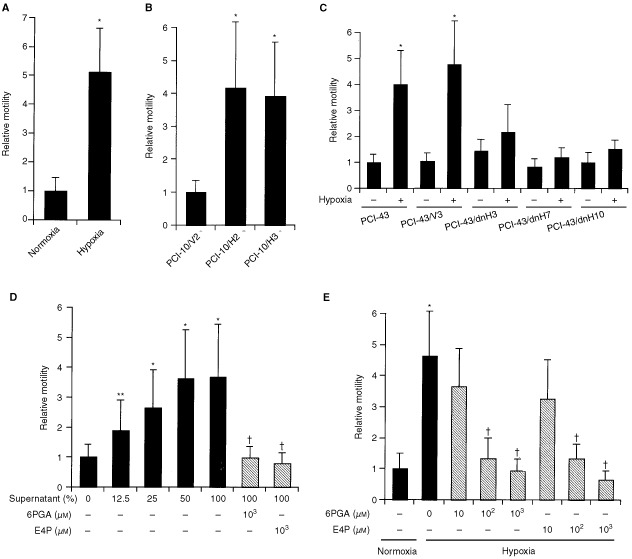
Random motility: Data present the mean±s.d. of three different experiments. (**A**) Random motility of the PCI-10 cells under hypoxic and normoxic conditions. *P<0.01; significantly different compared with the relative motility of the PCI-10 cells under normoxic conditions. (**B**) Random motility of the HIF-1α-transfectants under non-hypoxic conditions. *P<0.01; significantly different compared with the vector control. (**C**) Random motility of the dnHIF-1α-transfectants and the vector-transfectant under hypoxic and non-hypoxic conditions. *P<0.05; significantly different compared with the relative motility of the PCI-43 cells under normoxic conditions. (**D**) Random motility of PCI-10 cells under normoxic conditions in the presence or absence of the supernatant of PCI-10 cells cultured under hypoxic conditions. *P<0.01, **P<0.05; significantly different compared with the relative motility of PCI-10 cells under normoxic conditions in the absence of supernatant. †*P*<0.05; significantly different compared with the relative motility of PCI-10 cells in the presence of 100% supernatant. (**E**) Random motility of the PCI-10 cells under hypoxic conditions in the presence of AMF/PHI-inhibitors. *P<0.01; significantly different compared with the relative motility of PCI-10 cells under normoxic conditions. †*P*<0.01; significantly different compared with the relative motility of PCI-10 cells under normoxic conditions in the absence of inhibitor.

As shown in Figure 3A

, the random motility of PCI-10 cells increased under hypoxic conditions by more than four-fold compared with that under non-hypoxic conditions. Furthermore, the random motility of the HIF-1α-transfectants under non-hypoxic conditions was higher than that of the vector-transfectant (Figure 3B). Figure 3C shows the random motility of the dnHIF-1α-transfectants under hypoxic and non-hypoxic conditions. The random motility of the vector-transfectant but not of the dnHIF-1α-transfectants was enhanced under hypoxic conditions. These results in combination with the changes in the AMF/PHI/NL mRNA expression suggested that the random motility was closely correlated with the levels of AMF/PHI/NL mRNA expression.

Next, as AMF/PHI/NL has been reported to act in an autocrine fashion, we examined the effects of the supernatant of PCI-10 cells cultured under hypoxic conditions on their motility. Figure 3D shows that the supernatant enhanced the random motility of PCI-10 cells under non-hypoxic conditions in a dose-dependent manner, indicating that the random motility-stimulating activity in the supernatant stimulated it in an autocrine fashion. In order to determine whether the presumed random motility-stimulating factor in the supernatant was identical to AMF/PHI/NL, we examined the effects of two carbohydrate phosphates, 6-phosphogluconic acid (6PGA) and erythrose 4-phosphate (E4P), which have been reported to inhibit the PHI enzymatic activity and the AMF-induced cell motility (Watanabe *et al*, 1996). Figure 3D shows that 6PGA and E4P almost completely abrogated the motility-stimulating activity of the supernatant. Figure 3E shows that 6PGA and E4P directly reduced the random motility of PCI-10 cells enhanced under hypoxic conditions.

## DISCUSSION

A number of reports have demonstrated that the probability of distant metastases correlates with oxygen pressure in the tumour tissues (Gatenby *et al*, 1988; Brizel *et al*, 1996; Hockel *et al*, 1998; Rofstad, 2000), suggesting that hypoxia may promote metastatic potential. However, it remains poorly understood how hypoxia promotes the metastatic potential of tumour cells. In this study, we clearly demonstrated that hypoxia enhanced the AMF/PHI/NL mRNA expression in a variety of cancer cells and it also enhanced the random motility in an autocrine fashion. In accordance with our results, a recent report demonstrated that AMF/PHI/NL was identified as a hypoxia-inducible gene by a representational difference analysis using mRNA extracted from hypoxic and normoxic Capan-2, a human pancreatic cancer cell line (Yoon *et al*, 2001). However, it was not determined whether the enhanced expression of AMF/PHI/NL resulted in the enhanced motility. As hypoxia is frequently observed in tumour tissues *in vivo* (Guillemin and Krasnow, 1997; Blancher and Harris, 1998), we suspected that AMF/PHI/NL might be expressed in various cancer cells *in vivo*. In accordance with this speculation, previous reports demonstrated that the expression of AMF/PHI/NL was found in human colorectal, bladder, oesophageal and gastric cancers and that the expression of AMF/PHI/NL correlated with the disease progression (Watanabe *et al*, 1991; Nabi *et al*, 1992). Furthermore, we found that AMF/PHI/NL was expressed also in the tumour cells of human pancreatic cancers *in vivo* (data not shown). Recently we have reported that most pancreatic cancer cells, which are known to show high invasiveness and high metastatic potential *in vivo*, over-expressed HIF-1α proteins constitutively (Akakura *et al*, 2001). Those results in combination with the findings demonstrated in this study suggest that high expression of AMF/PHI/NL may be attributable to the high invasiveness and high metastatic potential of pancreatic cancers.

It is well-known that metastasis requires coordinated activation of various factors involved in proliferation, motility, cell-to-cell and cell-to-substrate contacts, degradation of extracellular matrix, inhibition of apoptosis, and adaptation to an inappropriate tissue environment (Poste and Fidler, 1980; Liotta *et al*, 1986). Our DNA microarray study showed that mRNA expressions of various angiogenic factors and glycolytic enzymes were enhanced in hypoxia in accordance with the previous reports (Vaupel *et al*, 1989; Semenza, 2000). Increased expression of angiogenic factors under hypoxic conditions enhances the angiogenesis that supports the survival and growth of tumour cells in the metastatic sites as well as in the primary sites (Claffey and Robinson, 1996; Rofstad and Danielsen, 1999). Likewise increased expression of glycolytic enzymes supports the survival and growth of tumour cells under hypoxic conditions (Malhotra and Brosius, 1999). Accordingly, these angiogenic factors and glycolytic enzymes induced by hypoxia could enhance the metastasis in cooperation with AMF/PHI/NL. Namely, hypoxia promotes the infiltration of endothelial cells into tumour tissues in its inducing angiogenesis; the hypoxia may also induce the activation of various factors other than angiogenic factors and AMF/PHI/NL in cancer cells. We now search the possible metastasis-associated genes, which should have hypoxia-responsive elements (HRE) in the promoter region.

All together, our present results provide a new insight into the mechanisms and a possible means for control of metastasis. We now propose that the enhancement of metastatic potential may be one of hypoxic responses of tumour cells exposed to hypoxia. The findings of dominant-negative HIF-1α-transfectants suggest that the disruption of the HIF-1 pathway may be an effective treatment for metastasis, in addition to the treatment of primary tumours through the inhibition of various genes necessary for the growth and metastasis of tumour cells *in vivo*, in accordance with the previous report (Kung *et al*, 2000).
